# Angiotensin-Converting Enzymes Play a Dominant Role in Fertility

**DOI:** 10.3390/ijms141021071

**Published:** 2013-10-21

**Authors:** Pei-Pei Pan, Qi-Tao Zhan, Fang Le, Ying-Ming Zheng, Fan Jin

**Affiliations:** Department of Reproductive Endocrinology, Women’s Hospital, School of Medicine, Zhejiang University, 1 Xueshi Road, Hangzhou 310006, China; E-Mails: 21118206@zju.edu.cn (P.-P.P.); greamygirl@zju.edu.cn (Q.-T.Z.); lefang851021@126.com (F.L.); 20918528@zju.edu.cn (Y.-M.Z.)

**Keywords:** angiotensin-converting enzyme, fertility, metabolic syndrome, renin-angiotensin system, angiotensin-converting enzyme inhibitor

## Abstract

According to the World Health Organization, infertility, associated with metabolic syndrome, has become a global issue with a 10%–20% incidence worldwide. An accumulating body of evidence has shown that the renin–angiotensin system is involved in the fertility problems observed in some populations. Moreover, alterations in the expression of angiotensin-converting enzyme-1, angiotensin-converting enzyme-2, and angiotensin-converting enzyme-3 might be one of the most important mechanisms underlying both female and male infertility. However, as a pseudogene in humans, further studies are needed to explore whether the *abnormal angiotensin-converting enzyme-3* gene could result in the problems of human reproduction. In this review, the relationship between angiotensin-converting enzymes and fertile ability is summarized, and a new procedure for the treatment of infertility is discussed.

## 1. Introduction

Over the past two decades, there has been a striking increase in the number of people with metabolic syndrome (MetS) worldwide. MetS is a highly prevalent condition currently considered to be a constellation of metabolic abnormalities, including blood pressure elevation, abdominal obesity, impaired glucose metabolism and hyperglycemia associated with insulin resistance (IR) [1–3]. Recently, studies have demonstrated that reproductive pathological conditions are associated with MetS, such as polycystic ovarian syndrome (PCOS), hypogonadism and erectile dysfunction [1–5]. It was estimated that approcimately 9% of the world’s reproductive population, which corresponds to 72.4 million couples, experience fertility problems [6]. In women, ovulation disorders prevail across different races [7,8]. Polycystic ovary, which is the leading cause of anovulatory infertility, affects 5%–7% of women of reproductive age [9]. Evidence of enhanced renin–angiotensin system (RAS) activity in PCOS suggests an important correlation between the RAS and PCOS [10,11]. Studies on the expression of angiotensin-converting enzyme-1 (ACE1) and angiotensin-converting enzyme-2 (ACE2) in male infertility cases have been reported, and *Ace1*^−/−^ male mice have been found to be sterile [12]. Despite the sperm motility and fusing location of eggs generated in *Ace2*^−/−^ and *Ace3*^−/−^ mice, the male mice were slightly abnormal, and both knockouts proved to be fertile [13,14]. The abovementioned facts indicate that ACE1, ACE2, and angiotensin-converting enzyme-3 (ACE3) appear to be one of the possible mechanisms responsible for infertility. Furthermore, the ACE1 has become a promising target for the treatment of MetS, which increases the risk factors of infertility, such as obesity, IR and so on [15–17]. Some studies further pointed out that ACE1 inhibitors (ACEIs) have become first-line drugs for some fertile issues [18,19]. This review attempts to summarize and explore the relationship between the sterility and ACEs expression in the ovaries and testes.

## 2. Renin–Angiotensin System (RAS)

**Figure 1:**
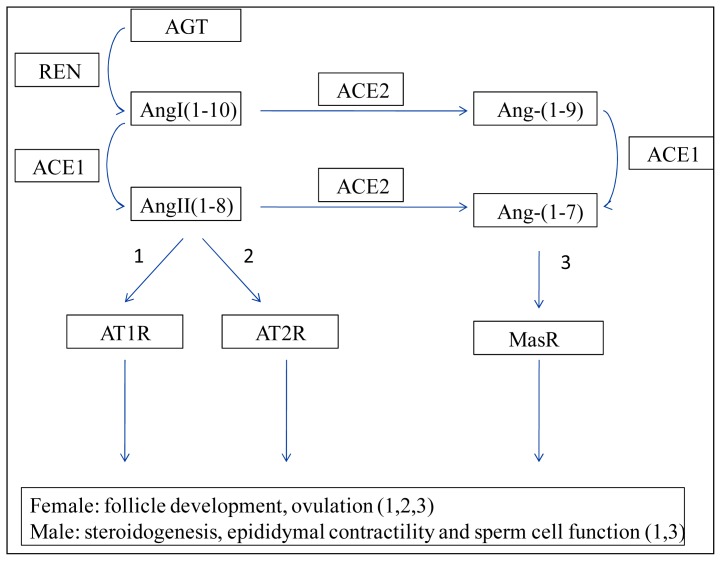
Schematic of the local renin–angiotensin system (RAS). AGT and REN are expressed in the liver and kidneys, respectively. Angiotensin peptides are produced through the action of REN, ACE1 and ACE2, all of which are peptidases. Digitals 1–3 refers to ACE1/AngII/AT1R, ACE1/AngII/AT2R and ACE2/Ang-(1–7)/MasR pathways, respectively. There are three main pathways in the ovarian RAS and two main pathways in the testis RAS. AngI (1–10), AngII (1–8), Ang-(1–9) and Ang-(1–7) are decapeptide, octapeptide, nonapeptide and heptapeptide, respectively. The peptide sequence of the AngI (1–10) is Arp-Arg-Val-Tyr-He-His-Pro-Phe-His-Leu. RAS: renin–angiotensin system; AGT: angiotensinogen; REN: renin; AngI: angiotensin I; AngII: angiotensin II; Ang-(1–9): angiotensin-(1–9); Ang-(1–7): angiotensin-(1–7); AT1R: angiotensin II type 1 receptor; AT2R: angiotensin II type 2 receptor; MasR: Mas receptor; ACE1: angiotensin-converting enzyme 1; ACE2: angiotensin-converting enzyme 2.

It is well acknowledged that the traditional RAS contains a system of finely tuned agonists and antagonists that balance blood pressure [20]. In recent years, attention has been focused on the physiological and pathophysiological studies of the human reproductive tract RAS. Classic components of the RAS have also been identified in the reproductive system, including in oocytes, granular cells, sperm cells, and Leydig cells [21,22]. Furthermore, the local RAS pathways, which are involved in reproductive events, have also been elucidated (Figure 1). ACE1, a key enzyme in the RAS, converts angiotensin I (AngI) into angiotensin II (AngII), which participates in female reproductive physiology via the AngII type 1 receptor (AT1R) and the AngII type 2 receptor (AT2R). In contrast, the functions of AngII in male reproductive events are stimulated by the AngII type 1 receptor (AT1R). ACE2, a homolog of ACE1, also emerges as a key factor in the regulation of the female and male reproductive performance that is mediated by angiotensin-(1–7) [Ang-(1–7)] [23–25]. Ang-(1–7), which is produced by ACE2, functions through the G protein-coupled receptor Mas [26,27]. To date, studies suggest that ACE3 might function in the testes of mice, rats, cows, and dogs, although ACE3 is not expressed in humans [28]. Collectively, the correct balance among the ACE1/AngII/AT1R, ACE1/AngII/AT2R and ACE2/Ang-(1–7)/Mas receptor (MasR) pathways is significant in female reproductive events, particularly follicle development, granulose-lutein (GL) cell apoptosis, ovulation, and the ACE2/Ang-(1–7)/Mas receptor axis [23,29–32]. In contrast, the ACE1/AngII/AT1R and the ACE2/Ang-(1–7)/Mas receptor pathways are involved in male fertile health, particularly steroidogenesis, epididymal contractility, and sperm cell function [21,26,33,34].

ACE1 is well recognized not only for its pivotal regulatory activities in cardiovascular homeostasis [35], but also for its influence on fertility. There are two distinct isoforms of ACE1: somatic ACE1 (sACE1) and germinal or testicular ACE1 (tACE1). These isoforms are transcribed from the same gene through the action of alternative promoters [36,37]. It has been determined that ACE1, which is a seminal fluid protein (SFP), protects sperm during and after transfer to females [38]. In females, ACE1 regulates the angiogenesis of ovarian endothelium and follicular growth; in contrast, in males, the sperm-migrating capability and binding ability to the zona pellucida (ZP) are affected by tACE1 [39]. Studies further demonstrate that only *tAce1*-knockout male mice are sterile, whereas *sAce1*-deficient male mice are fertile [40]. Moreover, sACE1 has been found to regularly express in human germ cells during fetal development, indicating that sACE1 may play a role in human germ cell development and ontogenesis [41–43].

**Figure 2:**
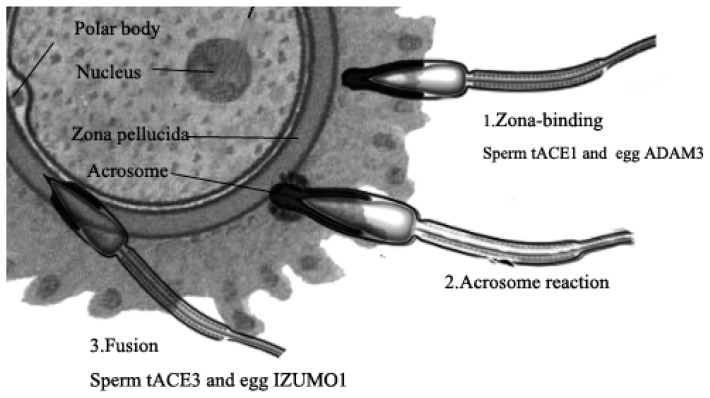
Mechanisms of sperm–egg interaction. tACE1 and ADAM3 are dispensable factors for the binding of sperm to the zona pellucida, whereas tACE3 and IZUMO1 play important roles in the fusion of gametes to sperm. ADAM: a disintegrin and metalloprotease; ZP: zona pellucida; tACE1: testis angiotensin-converting enzyme 1; tACE3: testis angiotensin-converting enzyme 3.

The *ACE2* gene, which was recently cloned, has an expression pattern that is restricted to endothelial cells in the heart and kidney, epithelial cells in the distal tubule of the kidney, and adult Leydig cells in the testis [36,44]. The full-length human ACE2 cDNA predicts an endothelium-bound carboxypeptidase of 805 amino acids, which has 42% homology with the *N*-terminal catalytic domain of ACE1 (Figure 2) and contains the following two domains: an amino-terminal catalytic domain and a carboxy-terminal domain [45]. The expression of ACE2 in the ovaries and testes suggests that this enzyme plays a regulatory role in steroidogenesis and thus affects germ cells and reproductive health.

In 2007, Rella *et al.* characterized the *ACE3* gene [28]. Unlike ACE1 and ACE2, ACE3 is not widely distributed. According to available data, ACE3 is only detected in the heart, testes, and embryos. ACE3 is expressed in mice, rats, cows, and dogs and lacks catalytic activity. Investigators attribute this lack of catalytic activity to a Gln substitution for the catalytic Glu in the putative zinc-binding motif. In humans, ACE3 contains a typical zinc-binding motif (HEMGH) that is similar to that of ACE1. However, no evidence was found that the *ACE3* gene is expressed, indicating that ACE3 is a pseudogene in humans [28]. Inoue and colleagues identified ACE3 as an IZUMO1-interacting protein in mouse sperm [14]. Through immunofluorescent staining, ACE3 was found to be located in the acrosomal cap area of fresh mouse sperm. After the acrosome reaction, ACE3 unexpectedly disappeared, and IZUMO1 remained in the sperm. IZUMO1 is considered the only sperm protein that has been proven to be essential for sperm–egg fusion.

## 3. Ovary ACEs

### 3.1. Ovary ACE1

In the 1980s, ACE1 was observed to be mostly expressed in large follicles in the ovaries. Immunoelectron microscopy analyses showed that ACE1 was distributed on the surface of follicular oocytes in a diffuse pattern and in the zona pellucida, which indicates its regulation during follicular development and oocyte maturation [46]. The intrafollicular injection of ACE1-forming AngII was found to prevent the expected atresia in the second-largest follicle, and these results imply that AngII plays a role in the regulation of follicular growth [47]. However, AngII, which is predominantly found in granulosa cells, is also involved in the development of atresia through the local induction of an increase in the follicular fluid androgen-to-estrogen ratio [48]. Furthermore, AngII is part of the intraovarian paracrine or autocrine control mechanism that takes place during the ovulatory process in the ovaries of pigs, rabbits, and cattle [49,50]. This effect may occur via AT2R because its specific antagonist, PD123319, reduces the AngII-induced ovulation [51]. The aforementioned facts imply that ACE1 indirectly influences the AngII-mediated development of follicles and ovulation. Another potential mechanism for the involvement of ACE1 in female fertility involves increased oxidative stress. It is well noted that reactive oxygen species can impair the pathophysiology of human reproduction [52–55]. One of the most important consequences of increased oxidative stress is the development of an inflammatory reaction. AngII has been reported to promote oxidative stress and to exert a pro-inflammatory effect through the activation of AT1R [56,57]. Thus, increased levels of ACE1, which produce excessive AngII, might damage the reproductive ability due to increased oxidative stress. However, captopril, which is an ACE1 inhibitor, does not affect ovulation in rats and rabbits, which suggests that the ACE1/AngII/angiotensin receptor pathway is not the only pathway that regulates ovulation and induces inflammation. Other pathways, such as the ACE2/Ang-(1–7)/Mas pathway, must therefore exist [58,59].

### 3.2. Ovary ACE2

Increasing data have demonstrated that ACE2 is present in human and rat ovaries [26,32]. The Ang-(1–7) peptides, which are produced by ACE2, are also located in several ovarian compartments and may be quantified in follicular fluid (FF) [27]. Gonadotropin induces changes in the ovarian expression of ACE2, Ang-(1–7), and the Mas receptor, which implies that ACE2 participates in ovarian physiology mediated by Ang-(1–7) [32]. Moreover, in addition to AngII, Ang-(1–7) has emerged as a key factor in the control of follicle deviation [25]. Ang-(1–7) and Mas, which are present in theca-interstitial cells, are able to stimulate ovarian steroidogenesis and thus modulate the ovarian physiological functions, such as follicular development, steroidogenesis, oocyte maturation, ovulation, and atresia [60]. The ACE2/Ang-(1–7)/Mas axis was recently verified to promote meiotic resumption, which is highly regulated by luteinizing hormone, likely as a gonadotrophin intermediate [61].

## 4. Testis ACEs

### 4.1. Testis ACE1

In the early 1980s, tACE1 was found to be absent in immature rats; however, this enzyme has been shown to develop with puberty, which indicates that its expression is under hormonal control [62]. Studies further show that tACE1 is exclusively expressed in developing spermatids and mature spermatozoa and it is localized in spermatid heads, residual bodies, and the cytoplasmic droplets of epididymal sperm [63,64]. Although tACE1 mRNA was found in spermatocytes, tACE1 protein was first present in post-meiotic step 3 spermatids and increased rapidly during further differentiation [43]. Nikolaeva *et al.* developed a very quantitative assay of tACE1 expression on human spermatozoa [65]. During the different phases of fertilization, the level of tACE1 expression on the sperm surface differed, which can dictate its role on reproduction. Therefore, it might be a new and useful tool for us to understand the roles of tACE1 and assess the reproductive ability. Moreover, the ACE1 found in seminal plasma is secreted or sloughed off from the prostate and epididymis [66]. *Ace1*^−/−^ mice exhibit impaired male fertility, and this impairment is rescued by the introduction of tACE1 into germ cells, which suggests that tACE1 plays a crucial role in male reproduction [12].

Many studies have reported the implication of tACE1 in capacitation. Mammalian spermatozoa must undergo a maturation process known as capacitation and a morphological change called an acrosome reaction before successful fertilization. During capacitation, sperm membranes are modified by the epididymal proteins located on their surface, and this is a crucial step to ensure successful sperm–egg interactions [67,68]. During epididymal passage, ACE1 minimizes the sperm motility by mediating the translocation of ADAM3 (Figure 2) [69]. ADAM family members, including a disintegrin and a metalloprotease, are required for normal mouse fertility [70]. Under capacitation conditions, evidence demonstrates that ACE1 is released from human spermatozoa *in vitro* and that this release is independent of the acrosome reaction [42,71]. Before binding to an egg ZP, spermatozoa adhere to the oviduct epithelium. Adherent spermatozoa may be released through the membrane tACE1. A portion of tACE1 is released from spermatozoa during capacitation, whereas other portions of tACE1 may be released during the sperm passage up the female reproductive tract to increase its binding capacity to the ZP [72]. In addition to its important role in capacitation, tACE1 has also been shown to participate in egg–sperm fusion. It was recently reported that tACE1 exhibits glycosylphosphatidylinositol (GPI)-anchored protein releasing activity (GPIase activity), and that this activity is identical to that of phosphatidylinositol-specific phospholipase (PI-PLC). Previous studies have demonstrated that the egg-binding deficiency of *Ace1*-knockout sperm can be rescued by peptidase-inactivated (inactivate the ability to cleave small peptides, such as AngI and Ang II) mutant ACE1 and PI-PLC, which implies that tACE1 plays a crucial role in fertilization through this activity [73,74]. However, many reports argued that the ACE1 does not possess considerable GPIase activity [75,76]. Leisle *et al.* [76] used multiple species of sACE1, porcine brush-border membrane and MDCK cells, while Kondoh *et al.* [77] utilized tACE1, HEK293 cells and Hela cells. And the differences between the studies of Fuchs *et al.* [75] and Kondoh *et al.* [73] might own to other intracellular factors with GPIase activity. Kondoh and colleagues further demonstrated that a set of glycans modulate the GPIase activity of ACE1 [78].

### 4.2. Testis ACE2

In the male reproductive tract, ACE2 is selectively expressed by adult Leydig cells in the testis. In addition, the ACE2-producing Ang-(1–7) and its receptor Mas have also been detected in the testis, and these are mainly located in the interstitial compartment and cytoplasm of the Leydig cells [26]. Reis *et al.* further demonstrated the strong influence of ACE2 in the male reproductive system by showing that humans with severe spermatogenesis impairment have lower levels of ACE2, Ang-(1–7), and Mas compared with fertile subjects [26]. Because the sex steroid hormone is one of the major products secreted from Leydig cells, it is suggested that ACE2 participates in the modulation of spermatogenesis. In contrast to *Ace1*^−/−^ male mice, which display significantly reduced fertility, both male and female *Ace2*-null mice are fertile [13], which suggests that the rescue mechanisms may be regulated by other reproduction-related proteins in the testis, such as tACE1 and ACE3. Moreover, there is evidence that the testis weight is markedly reduced in Mas-deficient mice [77]. Therefore, substantial evidence implies that ACE2 regulates spermatogenesis.

### 4.3. Testis ACE3

Similarly to tACE1, tACE3 is an IZUMO1-associated protein (Figure 2). IZUMO1, which is a novel sperm-specific protein with essential factors, is located in sperm–egg fusions in mice. *Izumo*^−/−^ males are infertile despite their normal mating behavior, ejaculation, and sperm motility [79]. However, *Ace3*^−/−^ mice are healthy and fertile and exhibit only slight mislocalization of IZUMO1-positive sperm compare with control mice [14]. These results suggest that the characteristic binding nature of tACE3 to IZUMO1 is not required for the fertilization of eggs by sperm.

## 5. Sterility of MetS

Besides the impaired glucose metabolism, dyslipidaemia and hypertension of MetS, sterility of women and men is also associated with MetS. In females, an aberrant ovarian RAS can result in the development of several gynecological diseases such as PCOS, the patients of which are more vulnerable to MetS [80]. PCOS is an ovulation disorder that causes impaired fecundity in females. Genetic studies further demonstrate that polymorphisms in *Ace1* are related to the risk factors for PCOS. Jia and his team proposed that *Ace1* insertion/deletion (I/D) polymorphisms are associated with an increased risk for PCOS [81]. The D allele, which is found in approximately 55% of the population, is associated with increased ACE1 activity [82]. A study further proposed that the *Ace1* DD genotype is related to increased IR in women with PCOS [83,84]. Moreover, PCOS is a common and complex disease with common features of hyperinsulinemia and IR. In addition to its effect on obesity and diabetes, abnormal insulin signaling has been linked to adverse pregnancy outcomes because it affects the female hypothalamic–pituitary–gonadal axis [85]. Accordingly, insulin-sensitizing drugs appear to enhance spontaneous ovulation and pregnancy rates [86].

In males, hypogonadism, erectile dysfunction and psychological disturbances are also often comorbid with MetS [1,2]. Several studies point to an increased likelihood of sperm disorders (oligozoospermia or azoospermia) and male infertility among overweight men [87,88]. Riera-Fortuny *et al.* found that type and grade of obesity correlated with the genotypes of the *ACE1* gene I/D polymorphism in subjects with coronary heart disease of MetS [89]. There is a significant correlation between hypertension, with more fragmented/abnormal sperm DNA, which is hypothesized that hypertension altered vascular status by enhancing reactive oxygen species (ROS) generation and limited antioxidant defence within the testes [90,91]. Furthermore, the levels of ROS are under the regulation of ACE1 and ACE2, activated by ACE1and attenuated by ACE2 [92,93].

## 6. Therapy for Infertility

Drugs inhibiting the RAS have shown benefits against multiple components of the MetS, indirectly ameliorate the reproductive health. Accumulating data indicate that ACE1 is a potential contributor to IR, which plays a crucial role in the pathogenesis of PCOS. Thus, treatment with the ACEI temocapril has been employed for females with IR, and this treatment improves their insulin sensitivity, which results in a favorable maternal and fetal outcome [19,94]. IR and hyperinsulinemia are implicated in the infertility of obese patients. In response to the stimulation of insulin, the serum levels of androgens are increased, and the synthesis of sex hormone binding-globulin (SHBG), which is the carrier protein for sex steroid hormones, decreases. In addition, adipose tissues store an excess amount of sex steroids, which could raise the plasma levels of androgens. The above mechanisms might lead to female infertility by impairing the ovulatory capacity of the ovaries [95]. Because ACEIs reduced the level of AngII, these drugs might downregulate insulin sensitivity not only by altering the insulin signaling pathways but also by diminishing the blood flow to muscles [96,97].

Despite the controversial study results, there are data supporting the use of ACEIs as effective drugs for the management of infertile men with idiopathic oligospermia, because of its beneficial effect on the sperm number, motility and morphology [18,98]. ACEIs exert beneficial effects on the sperm quantity and quality by blocking the conversion of bradykinin in the related kallikrein–kinin system into inactive peptides [99]. The accumulated bradykinin activates Sertoli cell function, regulates spermatogenesis, and leads to the maturation of spermatozoa [100]. However, some previous studies have not found any great improvement in the sperm quantity and quality after treatment with ACEIs, partially if a different dose of ACEIs is used [101].

To date, all of the drugs that target the RAS, including ACEIs and antagonists of angiotensin receptors, aim to decrease the RAS function. ACE2 may serve as a novel therapeutic component of the RAS that, if activated, could treat hypertension, IR and obesity of the MetS and other relative comorbid disease, such as infertility. A further understanding of the relationship between the ACEs and the sterility with or without MetS at specific cells may be an effective single therapy against infertility. In addition, dietary manipulations and sustainable strategies for weight loss benefit body composition and improve insulin regulation, which may ultimately treat specific features of MetS and improve the fertility.

## 7. Conclusions

As shown in this review, infertility is associated with MetS, risk factors of which might impair the reproduction. Moreover, there is no doubt that ACE1 and ACE2 have gained recognition as significant regulators of the physiology and pathology of the reproductive system. The sperm–egg fusion process is associated with the ADAMs-associated protein ACE1 and IZUMO1-interacting protein ACE3. However, the fertility of *Ace1*/*Ace2*, *Ace1*/*Ace3*, and *Ace2*/*Ace3* double mutants has not been addressed. If these double-mutant mouse models are generated, the association between ACEs and the cause of infertility could be elucidated more clearly. Moreover, ACEIs have become first-line drugs for the management of PCOS-related IR in infertile females and idiopathic oligozoosperm in males, although some controversial results have been observed. Thus, the aforementioned findings require confirmation in larger multicenter studies.
